# Family composition, income, and healthy diet in rural China: evidence from three provinces

**DOI:** 10.3389/fnut.2025.1608024

**Published:** 2025-06-04

**Authors:** Minda Yang, Nimra Amar, Longqiang Zhao, Cong Pan

**Affiliations:** College of Economics and Management, Huazhong Agricultural University, Wuhan, China

**Keywords:** healthy diet, sustainable food consumption, family composition, income, rural China

## Abstract

A healthy diet is essential for public health and plays a central role in sustainable food systems. This paper investigates the impact of family composition and income on dietary health within rural households in China, analyzing how these factors correlate with dietary diversity and quality. Utilizing a 3-day food consumption record data across three provinces, we apply ordinary least squares regression to explore the relationships with three key dietary health indicators: the Entropy Index of dietary diversity (E), the Chinese Food Pagoda Score (CFPS), and the Chinese Healthy Eating Index (CHEI). Our findings indicate that higher household income significantly improves all measures of dietary health, affirming the critical role of economic factors in achieving a nutritious diet. In contrast, a higher proportion of elderly individuals within households is associated with poorer dietary outcomes, suggesting specific challenges in meeting the nutritional needs of aging populations. These findings offer insights into how demographic and income factors shape dietary practices, contributing to discussions on sustainable food consumption in developing regions. This research contributes to the understanding of dietary health dynamics in rural China and supports broader efforts to attain sustainable development goals related to health and wellbeing.

## 1 Introduction

Promoting dietary health is essential for achieving sustainable food systems, as it supports both environmental goals and public health. According to the World Health Organization, a healthy diet should comprise balanced, diverse, and sufficient nutrient intake predominantly from plant-based sources, complemented by moderate animal-source foods and limited processed products high in sugars, fats, and salt (1). Proper food intake and nutrition enhance cognitive function, decrease vulnerability to diseases, and boost productivity (2, 3). These benefits are central to the United Nations' Sustainable Development Goals, particularly Goal 2: Zero Hunger, which seeks to eliminate hunger and ensure universal access to healthy food, and Goal 3: Good Health and Wellbeing, aimed at promoting wellbeing for all at all ages. Despite these ambitious goals, the realization of a healthy diet in many developing countries and rural areas is far from being achieved (4–6). This global framework sets a crucial context for analyzing the specific conditions in rural China, where dietary health is intricately linked to economic and social factors.

The income level of rural residents in China continues to increase, but the demographic structure has changed and the population is aging seriously, which may further affect the healthy diet of rural households. Previous studies have explored the influencing factors of healthy diet. First, income increase is closely related to a healthy diet. As household income rises, typically so does the capacity to purchase a variety of foods, particularly those that are nutrient-dense yet often more costly, such as quality proteins (7–9). Empirical studies frequently correlate higher income levels with increased food diversity and improved diet quality (10–12). Second, an aging population presents unique healthy diet challenges. Elderly individuals have different dietary requirements, often necessitating increased nutrient-dense foods and fewer calories (13, 14). Moreover, age-related changes in taste, dental health, and digestion can shift dietary preferences and requirements, potentially leading to a decreased healthy diet (15–17). Third, labor force transfer, particularly from agricultural work to industrial or service sectors, can significantly alter dietary habits. This transition often introduces rural residents to urban dietary patterns, which can be both beneficial and detrimental. While urbanization can increase access to diverse foods, it also raises the risk of adopting less healthy, calorie-dense diets (18, 19). Fourth, the transformation of family compositions has profound dietary implications. Previous literature has also highlighted the role of household composition in dietary choices, suggesting that the presence and proportion of different age groups within the household can significantly dictate food consumption patterns (20–22). In addition, factors such as dietary knowledge, educational level of dietary decision-makers, and development of the food market are also considered important factors affecting healthy diet (11, 23, 24).

Although previous studies have focused on the impact of income and population structure on healthy diet. Studies have illustrated that increased income generally leads to improved dietary diversity and quality (25, 26). Similarly, the composition of a household has been shown to significantly influence diet patterns (27–29). However, there is little research on the impact of rural population aging and increasing income of farmers on the healthy diet of rural households. It is still unclear how the family composition especially aging of rural households affect healthy diet. Meanwhile, in the context of population aging, the impact of income levels of rural residents on healthy diets needs to be further explored.

The objective of this study is to estimate the impact of family structure and income on household healthy diet in rural China. There are three main reasons for selecting China as the research subject. Firstly, since the 20th century, China has experienced rapid economic growth, and the income of rural residents in China has also been significantly increased (30, 31). However, China also faces a serious problem of aging population. According to China's national population census in 2020, 13.5% of the populations is 65 years old and above. The aging problem is more severe in rural areas of China, with the proportion of people aged 65 and above reaching 17.7%. Secondly, given the rapid economic growth and increasingly severe aging population in China, it is essential to investigate how family composition and income affect healthy diets in rural households. This exploration will enhance our comprehension of healthy eating habits in rural China and help in devising suitable strategies. Third, extant studies indicate that dietary quality among rural Chinese households remains markedly below prescribed standards. In 2022, the mean dietary diversity score was only 3.9 out of 10, with fewer than one quarter of households meeting the minimum diversity threshold (11). Likewise, the Healthy Eating Index averaged 60 on a 100-point scale, substantially trailing urban counterparts (24). There remains considerable room for improvement in rural diet quality, as undernutrition and micronutrient gaps persist in many regions (80). The persistent insufficiency in the intake of nutrient-dense foods coupled with low dietary diversity underscores the substantial scope for improving nutritional status in rural areas (81). Addressing these dietary deficiencies is therefore critical both for improving rural wellbeing and for advancing China's progress toward the United Nations Sustainable Development Goals, particularly SDG 2 on ending hunger and malnutrition, and SDG 3 on ensuring healthy lives for all.

To achieve this goal, we empirically examine whether family composition and income affect healthy diet in rural China. The dataset used was from the household food consumption survey of 1,080 smallholder farmers carried out in rural areas of Jilin, Shandong, and Hubei Provinces, China in 2022. A 3-day dietary record method was utilized to gather household food consumption data from sample households, offering a dependable dataset for assessing household healthy diet. Firstly, we employed an ordinary least squares regression to estimate the impact of family composition and income on household healthy diet. Secondly, we estimated the heterogeneity of the impact of family composition and income on healthy diet. Thirdly, we estimate the impact of family composition and income on food consumption among different food groups. Fourthly, we analyze and explore the potential future of a healthy diet in China using the findings of this paper, taking into account income growth rates and population trends in China. Finally, we conducted robustness testing using machine learning methods. Further validated the changes in China's healthy diet with income and population aging.

Our study makes several contributions. Firstly, this research enriches the literature of healthy diet. Although previous studies have shown that income consistently has a significant positive effect on healthy diet (32, 33), most have focused on the impact of family composition and income separately on healthy diet. Specifically, the impact of population aging on healthy diet has worse significance for low-income rural households. Secondly, this study advances research on the healthy diet of Chinese rural households. Being the largest developing country, the nutritional status of Chinese rural residents has a significant impact on the global economy. The study specifically focuses on the healthy diet of Chinese households and recently conducted a comprehensive socioeconomic survey in three provinces in China. Additionally, food consumption data for rural residents was collected over a period of three consecutive days. The current nutritional status of Chinese rural residents has been uncovered, serving as a reference for future research.

## 2 Data and descriptive statistics

### 2.1 Data collection

The data for this study was collected in July 2022 across three provinces in China: Jilin, Shandong, and Hubei. Before the main survey, we conducted a pilot study in July 2021 in Dalian, Liaoning Province; feedback from this exercise led to several iterative revisions of the questionnaire. To ensure a representative sample reflecting diverse socioeconomic and agricultural conditions, we employed a stratified random sampling method. The provincial sampling framework is based on the per capita disposable income level of rural residents in 2021, according to the National Bureau of Statistics (Shandong, Hubei, and Jilin ranked 8th, 14th, and 19th respectively), and on geographical location characteristics (representing the eastern, central, and northeastern regions, respectively). It comprehensively selects sample provinces to cover regional development gradient differences. The stratification criteria included population size, economic status, and agricultural output of the regions. Within each province, four counties were selected. From each county, three townships were chosen, and subsequently, three villages were identified within each township. In each selected village, a comprehensive list of rural households was used as the sampling frame. From this list, 10 households were randomly selected per village, resulting in a total of 1,080 sample households. This process culminated in a robust sample of households spread across 108 villages in 36 townships of 12 counties across the three studied provinces. The geographical distribution of the selected provinces and counties is shown in Appendix Figure A1. All enumerators, senior undergraduates and graduate students in agricultural economics, underwent rigorous pre-field training that covered interview protocols, portion-size estimation, and data entry with built-in range checks. The primary data collection involved face-to-face interviews with household heads or main food decision-makers. Immediately after each interview, enumerators performed multiple rounds of on-site and end-of-day consistency checks; any discrepancies flagged by the survey software were resolved through prompt call-backs or supervisor verification. These combined procedures, including pre-survey piloting, intensive enumerator training, and systematic post-survey validation, ensure the reliability and high quality of the dataset. The primary data collection involved face-to-face interviews with household decision makers or those responsible for household food decisions.

The dataset includes surveys of rural households and villages. The household survey collected extensive data on the socioeconomic status of each family member, household farming conditions, non-agricultural economic activities, and other socio-economic details such as household assets. Specifically, the dataset documented household food consumption details for three consecutive days. The 3-day household food consumption record data includes all food consumed at home. To mitigate bias from eating-away-from-home, the survey collected the count of meals eaten at home by family members and non-family members during the survey period (34). In addition, the village questionnaire contains essential details about the village (e.g., population, agricultural production status), basic information regarding village officials (e.g., age, education level, years of service), the status of the village food market, and other socio-economic features of the village.

### 2.2 Measure of key variables

#### 2.2.1 Healthy diet

A healthy diet is crucial for maintaining overall health and wellbeing, as it provides essential nutrients, supports immune function, and reduces the risk of chronic diseases (35, 82). The dependent variables in this study are designed to capture various dimensions of healthy diet among rural households in China. These include the Entropy index (also called the Shannon index) of dietary diversity (EI), the Chinese Food Pagoda Score (CFPS), and the Chinese Healthy Eating Index (CHEI). To avoid including certain food groups of lower nutritional quality that may contribute to dietary diversity without necessarily improving dietary quality, such as sugar, sweets, and soft drinks, twelve primary food groups were selected to calculate three variables. Each variable provides a distinct perspective on dietary intake and its alignment with nutritional recommendations.

Following Liu et al. (36), El gauges the evenness of intake across twelve food groups (cereals; tubers; dried legumes; vegetables; fungi/algae; fruits; nuts/seeds; meat; poultry; dairy; eggs; aquatic products): $EI=-\overset{12}{\underset{i=1}{\sum }}w_{i}\ln\left(w_{i}\right)$, where *w*_*i*_ is the proportion of total edible weight contributed by group *i*. Values run from 0 (complete monotony) to ln(12) = 2.48 (perfect homogeneity). To assess adherence to the Chinese Dietary Pagoda (2016) we aggregate the eight groups into the Pagoda's eight core categories-(i) grains/tubers/legumes, (ii) vegetables, (iii) fruits, (iv) meat and poultry, (v) eggs, (vi) aquatic products, (vii) dairy, and (viii) nuts/seeds—and apply the Huang and Tian (37) three tier rule:


$$\begin{array}{l} s_{j}=\{\begin{array}{cc} 1 & \text{ if }L_{j}\leq {\text{intake}}_{j}\leq U_{j},\\ 0.5 & {\text{if }|intake}_{j}-L_{j}|\leq 0.5L_{j}\;or\;|{\text{intake}}_{j}-U_{j}|\leq 0.5U_{j}\\ 0 & otherwise, \end{array}, \end{array}$$

where *L*_*j*_ and *U*_*j*_ are the Pagoda's lower and upper bounds for category *j*. The composite index $\text{CFPS}=\overset{8}{\underset{j=1}{\sum }}s_{j},\;0\leq \text{CFPS}\leq 8$, rises as consumption converges on guideline ranges. CHEI evaluates overall diet quality, again using the same twelve food groups. Each group *k* is classified either as an adequacy component (encouraged foods) or a limitation component (foods to curb). Every component carries the same maximum weight *W*_*k*_ = 5 points, so the total index ranges from 0 to 60. Adequacy scoring $c_{k}^{A}=\text{min}\left(\frac{{\text{intake}}_{k}}{R_{k}},1\right)\times 5$, where *R*_*k*_ is the recommended intake for component *k*. Limitation scoring $c_{k}^{L}=$ $\{\begin{array}{cc} 5, & {\text{ if intake }}_{k}\leq L_{k},\\ \left[1-\frac{{\text{intake}}_{k}-L_{k}}{U_{k}-L_{k}}\right]\times 5, & \text{if }L_{k}<{\text{intake}}_{k}<U_{k}\\ 0, & {\text{ if intake}}_{k}>U_{k}, \end{array}$, with *L*_*k*_ denoting the ideal upper limit and *U*_*k*_ the cut-off for zero points. Aggregation $\text{CHEI}=\overset{12}{\underset{k=1}{\sum }}c_{k},\;0\leq \text{CHEI}\leq 60$. Higher CHEI values indicate diets that are simultaneously nutrient-dense (adequacy components near or above targets) and restrained in less desirable items (limitation components kept below upper limits).

#### 2.2.2 Family composition and income

The independent variables of interest in this study are family composition and income. Family composition is quantified through three distinct proportions within each household: the proportion of members under 18 years old, the proportion aged 18 to 65, and the proportion aged 65 and above (38, 39). Family members under 18 are classified as minors. Those between 18 and 65 are deemed capable of working. Family members 65 and older are considered elderly. Minors and the elderly typically have limited work capacity, unstable income sources, and are also in need of a nutritious diet to maintain their physical wellbeing (40, 41). This segmentation allows for a detailed analysis of how healthy diet are influenced by the demographic structure of the household, recognizing different economic roles across age groups. Income is measured as the annual per capita income of households, expressed in Chinese Yuan. This economic indicator is crucial for assessing the affordability and accessibility of diverse and nutritious foods, providing insight into how financial resources impact dietary practices (42, 43).

#### 2.2.3 Other control variables

In addition to family composition and income, most studies share a set of control variables in the influence of household socioeconomic characteristics on healthy diet (24, 37). According to existing studies, the controls in this study are categorized into three groups: food decision-maker controls, household controls, and village controls (9, 44). Individual controls capture the characteristics of food decision maker, including gender, marital status, education level and dietary knowledge (11, 24, 45). Dietary knowledge was assessed using questions based on the China Health and Nutrition Survey (CHNS). For each question, respondents selected from the options “agree,” “disagree,” or “unknown.” A score of 1 was assigned for a correct answer and 0 for an incorrect or unknown response, and these scores were used to construct a dietary knowledge index (DKI). The DKI ranges from 0 to 9, with higher scores indicating better dietary knowledge. At the household level, we included the amount of pension the household receives, income from agriculture and off-farm work, distance to the nearest market, land area available to the household, crop diversity, household size, and total family property to show their influence on diet quality in rural China and comparable settings (11, 24, 46). Finally, village-level controls such as population size, distance to the township center, food-market density, and average income serve as proxies for the broader socio-economic environment that influences food availability, price dispersion, and infrastructure quality (11, 37, 45). To control for unobserved regional heterogeneity, county dummy variables are included in the estimation.

### 2.3 Descriptive statistics

The descriptive analysis presented in Table 1 elucidates the defining characteristics of the variables employed in this study. The dependent variables, including the Entropy Index of Dietary Diversity (mean = 1.26), the Chinese Food Pagoda Score (mean = 1.99), and the Chinese Healthy Eating Index (mean = 23.15), indicate the range of dietary health within the sample. The values of three variables indicate that the overall quality of the diet among rural residents is typically low. The interested independent variables of family composition are broken down by age groups, with the proportion of household members under 18 (mean = 9.17%), aged 18– 65 (mean = 63.46%), and over 65 (mean = 26.61%), highlighting demographic diversity. Income is reported as annual per capita household income (mean = 2.53 10,000 Yuan), reflecting the economic status of the sampled rural households.

**Table 1:** Descriptive analysis and definition of variables.

**Variables**	**Definition**	**Mean**	**SD**
E	Entropy index of dietary diversity (0–2.48)	1.26	0.30
CFPS	China Food Pagoda Score (0–8)	1.99	1.04
CHEI	Chinese healthy eating index (0–60)	23.15	6.22
Age < 18	People aged under 18 ratio of household members (%)	9.17	15.36
Age 18–65	People aged 18–65 ratio of household members (%)	63.46	37.35
Age ≥ 65	People aged 65 and above ratio of household members (%)	26.61	39.12
Income	Per capita household income (ten thousand Yuan)	2.53	2.51
**Food decision maker controls**			
Gender	1 if female and 0 if male	0.78	0.41
Married	1 if married and 0 if otherwise	0.91	0.28
Education	1 if college degree or above and 0 if otherwise	0.03	0.17
DKI	Dietary knowledge index	7.11	1.52
**Household controls**			
Pension	Pension amount (10,000 Yuan)	0.19	0.51
Ln(pension)		0.13	0.26
Off farm work	Off-farm employment proportion of household members	0.35	0.32
Distance	Average distance to free market where residences go most often to buy food (km)	3.02	3.80
Ln(distance)		1.07	0.77
Land	Per capita household land area (ha)	0.34	0.44
Ln(land)		0.25	0.25
Crop diversity	Households' number of crop productions	5.66	2.73
Household size	Number of household members	3.16	1.49
Property	Family property amount (ten thousand Yuan)	16.09	36.52
**Village controls**			
Population	Total resident population of village	900.95	616.59
Village distance	Distance from village to township (km)	5.45	5.77
Food market density	Number of food shops within three kilometers of the village	13.73	10.94
Village Income	Village per capita income (ten thousand Yuan)	1.63	0.61
Ln(Village income)		0.94	0.23
Village land	Total village land area (ha)	322.73	313.15
Ln(Village land)		7.52	1.27

Food decision-maker controls include gender (78% female), marital status (91% married), and education levels (3% with college degree or above). Household controls provide insight into the socio-economic conditions, such as pension amounts, agricultural income, off-farm work, market distance, land area, crop diversity, household size, and property amount. Village controls capture the broader community context with variables like village population, distance to township, food market density, and village per capital income.

**Figure 1:**
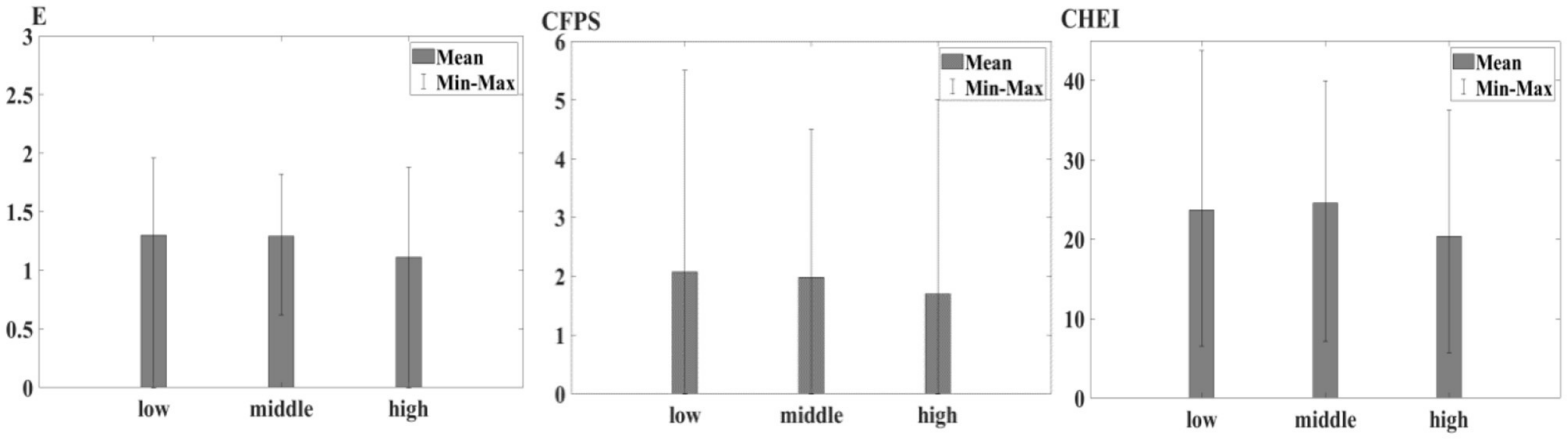
Healthy diet among different elderly people proportion group.

Figure 1 illustrates the relationship between the proportion of elderly individuals (aged over 65) within rural households and various indices of healthy diet. The bar charts convey that households with a low proportion of elderly individuals display comparatively higher mean values on the dietary indices, indicating more favorable dietary conditions. As the proportion of elderly members transitions from low to high, there is a noticeable decrement in mean values across all healthy diet measures. The bars for the higher elderly proportion groups show reduced mean levels of the healthy diet indices, suggesting a potential correlation between an aging demographic and healthy diet.

**Figure 2:**
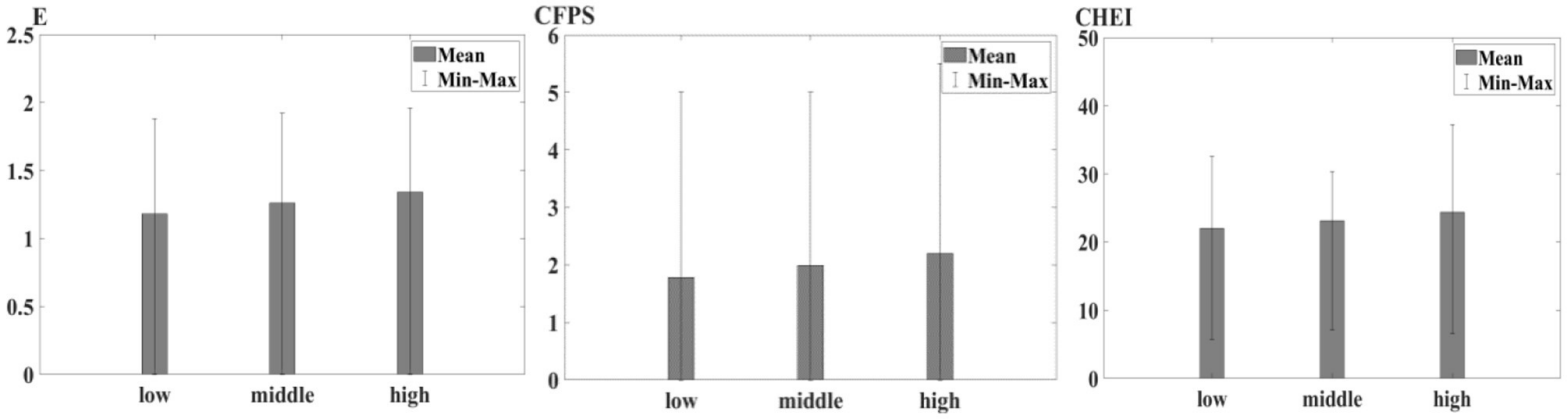
Healthy diet among different income group.

Figure 2 presents the relationship between household income levels and healthy diet across rural Chinese households. The bar charts compare the mean scores of healthy diets across three distinct income categories: low, middle, and high. Households in the low-income category demonstrate the lowest mean values for all healthy diet measures, indicating a correlation between limited financial resources and poorer dietary conditions. As household income increases to the middle-income bracket, there is an observable rise in the mean scores of all three dietary indices, suggesting an improvement in dietary quality and adherence to nutritional guidelines. This positive trend continues and is even more pronounced in the high-income category, which exhibits the highest mean values across the dietary indices, signaling a substantial association between increased financial capacity and better dietary outcomes.

## 3 Empirical model

### 3.1 Econometric model specification

To investigate the effects of family composition and income on household dietary health in rural China, a multivariate linear regression models were established and expressed as follows:


(1) $$\begin{array}{l} y_{i}=\alpha_{i}+\sum \beta_{i}ratio_{j}+\gamma_{i}income_{i}+\theta_{i}Z_{i}+\varepsilon_{i} \end{array}$$

Where *y*_*i*_ represents the dependent variables (Entropy Index of Dietary Diversity, Chinese Dietary Pagoda Score, and Chinese Healthy Eating Index) for the *i*^th^ household; *ratio*_*j*_ represents a set of independent variables that indicate the household demographic structure based on the proportion of various age groups; *income*_*i*_ represents the per capita family income for the *i*^th^ household; *Z*_*i*_ represents other control variables (food decision maker characteristics, household characteristics, and village characteristics); α_*i*_, β_*i*_, γ_*i*_, and θ_*i*_ are the coefficients to be estimated, and ε_*i*_ is the error term. The estimated parameter representing the proportion of family members aged over 65 years old will serve as a key metric for assessing the influence of population aging on healthy diet. In all regressions, standard errors are adjusted for clustering at the household level to account for potential correlations within the same household.

The empirical strategies for estimating the above equations are summarized as follows. First, 3-day food consumption record data can be used to calculate the household healthy diet. The use of several healthy diet indicators also assists in detecting the robustness of the main results. Second, ordinary least squares (OLS) regression is applied to estimate Equation 1 after considering the forms of the E, CFPS, and CHEI. Third, a series of heterogeneity analyses are conducted to explore how the relationships between family composition, income, and healthy diet vary across different observable household characteristics. We attempt to explore the heterogeneous effects of family composition on dietary diversity among rural households in different income quantiles. Then, the heterogeneous effects of family composition and income on healthy diet among rural households with different labor number, pension amount, food market density and land area are explored. Finally, a seemingly unrelated regressions (SUR) model is used to estimate the impact of family composition and income on household food consumption among different food groups.

### 3.2 Simulation of healthy diet trend in China

Based on the regression analysis previously described, we calculate the parameter β_3_, which quantifies the impact of population aging on healthy diet. Let γ_*i*_ denote the coefficient representing the effect of income growth on a healthy diet. Assuming the population aging in year *t* is *ratio*_*t*_ with an annual growth rate of *m*, the population aging can be modeled as: *ratio*_*t*_ = *ratio*_*t*−1_ × (1+*m*). Similarly, if the income in year *t* is *income*_*t*_, growing annually at rate *n*, it can be expressed as: *income*_*t*_ = *income*_*t*−1_ × (1 + *n*). Then, the healthy diet in year *t* can be expressed as follows:


(2) $$\begin{array}{l} {\;y}_{t}=\alpha_{i}+\beta_{3}ratio_{t}+\gamma_{i}income_{t} \end{array}$$

Where *y*_*t*_ represents healthy diet in year *t*; *ratio*_*t*_ and *income*_*t*_ represent population aging and income in year *t*, respectively; α_*i*_ represents the constant of the regression model; β_3_ presents the coefficient of population aging of the regression model; γ_*i*_ presents the coefficient of income of the regression model.

## 4 Results and discussion

### 4.1 Impact of family composition, income on healthy diet

Table 2 presents a comprehensive regression analysis assessing the impact of family composition and income on three key dietary health indicators: E, CFPS, and CHEI. The results indicate that the proportion of household members under 18 (Age < 18) has a positive effect on E, suggesting that younger household demographics are associated with a more diverse diet. A key finding is the significant influence of the proportion of elderly household members (Age ≥ 65) on all dietary health indicators. Specifically, the negative coefficients for this demographic segment across the E, CFPS, and CHEI suggest that an increased presence of older members is associated with lower dietary diversity and adherence to recommended dietary guidelines. The impact of the proportion of elderly household members variable can be explained by several factors prevalent in rural China. Older individuals may have specific dietary preferences or requirements, often associated with lower calorie needs and potentially more restricted diets due to health issues, which could lead to less varied food intake. Furthermore, this age group might be more entrenched in traditional eating habits, which do not always align with the modern nutritional guidelines captured by the CFPS and CHEI. Comparatively, existing literature highlights that older adults often face challenges such as decreased mobility, reduced income after retirement, and chronic health conditions, all of which can contribute to limited dietary diversity. For instance, Wetherill et al. (83) have found that reduced physical function can negatively impact the ability to purchase and prepare diverse foods, thereby affecting diet quality.

**Table 2:** Impact of family composition, income on healthy diet.

**Variable**	**E**	**CFPS**	**CHEI**
Age < 18	0.002^**^	−0.000	0.006
	(0.001)	(0.002)	(0.017)
Age ≥ 65	−0.001^**^	−0.002^*^	−0.016^**^
	(0.000)	(0.001)	(0.006)
Income	0.017^***^	0.025^*^	0.139^*^
	(0.004)	(0.013)	(0.081)
Gender	0.027	0.188^**^	0.933
	(0.025)	(0.073)	(0.564)
Married	0.031	−0.047	0.134
	(0.033)	(0.099)	(0.690)
Education	0.058	0.269	1.553
	(0.074)	(0.167)	(1.610)
DKI	0.012^*^	0.067^***^	0.329^***^
	(0.007)	(0.022)	(0.120)
Ln(pension)	0.109^***^	0.299^***^	2.399^***^
	(0.021)	(0.087)	(0.660)
Off farm work	−0.032	−0.015	−0.065
	(0.033)	(0.103)	(0.653)
Ln(distance)	0.002	−0.025	0.023
	(0.012)	(0.047)	(0.254)
Ln(land)	0.008	0.022	−0.055
	(0.015)	(0.060)	(0.351)
Crop diversity	0.000	0.025^**^	0.139^*^
	(0.003)	(0.011)	(0.080)
Household size	0.013	0.048	0.332
	(0.009)	(0.031)	(0.197)
Property	0.000	−0.000	−0.008
	(0.000)	(0.001)	(0.009)
Population	−0.000	−0.000	−0.000
	(0.000)	(0.000)	(0.001)
Village distance	−0.002	−0.008	−0.027
	(0.001)	(0.006)	(0.029)
Food market density	0.001	0.004	−0.005
	(0.002)	(0.005)	(0.034)
Ln(village income)	0.040	0.021	−0.720
	(0.058)	(0.197)	(1.165)
Ln(village land)	0.037^**^	0.072	0.311
	(0.015)	(0.051)	(0.376)
County FE	Yes	Yes	Yes
Constant	0.693^***^	1.064^**^	17.373^***^
	(0.135)	(0.477)	(3.425)
N	1079	1079	1079

Robust Standard errors in parentheses. ^*^ *p* < 0.10, ^**^ *p* < 0.05, ^***^ *p* < 0.01.

The variable of income stands out with a significant positive coefficient across all dietary indices, implying that increases in income are strongly correlated with improvements in dietary diversity (E) and adherence to dietary guidelines (CFPS and CHEI). This finding is consistent with economic theories that posit wealth as a primary enabler of access to a varied and nutritious diet (47). Additionally, previous research in similar socio-economic contexts indicates that financial constraints in retirement can lead to a reliance on staple foods over a diverse diet, which may compromise nutritional intake (48).

Other significant control variables include the pension income [Ln(pension)], which shows a strong positive relationship with healthy diet, indicating that retirement benefits may play a critical role in enabling older populations to afford a healthy diet (49, 50). The variable DKI possibly representing a diet knowledge index, shows a significant positive impact on all dietary health measures, reinforcing the importance of nutritional awareness in dietary choices (11). County-level fixed effects are controlled for, indicating that regional heterogeneity has been considered and suggesting that the impacts identified are robust across different areas.

### 4.2 Heterogeneity analysis

#### 4.2.1 Income

Table 3 presented the results of a heterogeneity analysis examining the differential impacts of income levels on the relationship between family composition and dietary health outcomes. The results indicate that income stratification yields distinct effects on dietary outcomes. For households with a higher proportion of members under 18 (Age < 18), the impact on E is positive across all income levels, with the effect size being the largest for high-income households. This suggests that higher income enhances the ability of families to provide diverse diets for younger members, which aligns with literature indicating that financial resources allow for greater food variety (51).

**Table 3:** Heterogeneity analysis based on income.

**Variable**	**E**			**CFPS**			**CHEI**		
	**Income**			**Income**			**Income**		
	**Low**	**Middle**	**High**	**Low**	**Middle**	**High**	**Low**	**Middle**	**High**
Age < 18	0.001	0.002	0.003	0.000	−0.002	0.001	−0.000	−0.019	0.051
	(0.001)	(0.002)	(0.002)	(0.004)	(0.006)	(0.006)	(0.023)	(0.040)	(0.035)
Ratio ≥ 65	−0.001^*^	−0.001^*^	0.000	−0.001	−0.003	0.000	−0.015^**^	−0.016	0.001
	(0.000)	(0.001)	(0.001)	(0.001)	(0.002)	(0.003)	(0.007)	(0.014)	(0.026)
Controlled	Yes	Yes	Yes	Yes	Yes	Yes	Yes	Yes	Yes
County FE	Yes	Yes	Yes	Yes	Yes	Yes	Yes	Yes	Yes
Constant	0.608^***^	0.904^***^	1.301^***^	1.291^**^	0.689	0.988	16.252^***^	19.241^***^	30.339^***^
	(0.170)	(0.282)	(0.331)	(0.568)	(1.002)	(1.759)	(3.214)	(6.209)	(9.561)
N	575	310	194	575	310	194	575	310	194

Robust Standard errors in parentheses. ^*^ *p* < 0.10, ^**^ *p* < 0.05, ^***^ *p* < 0.01.

Conversely, in the low-income group, the impact of the proportion of elderly household members (Age ≥ 65) on the E and CHEI is notably negative, suggesting that older household demographics with lower income are linked to reduced dietary diversity and compliance with dietary guidelines. Notably, the adverse effect on E and CHEI is less significant for high-income households, suggesting the mitigating influence of economic status on the dietary restrictions commonly experienced by the elderly. Previous studies have observed that increased financial capacity may offset some of the dietary limitations due to aging, such as the ability to afford specialized diets and health products (52).

#### 4.2.2 Labor number

Table 4 presents a heterogeneity analysis exploring the interaction between labor force size within rural households and the impact on dietary health outcomes in China. Labor force size, delineated as households with less than or equal to two members (≤2) and those with more than two members (>2), appears to moderate the relationship between family composition, income, and dietary health. For households with a higher proportion of members under 18, we observe a positive impact on E across labor force sizes. The effect is more pronounced in households with a larger labor force, potentially indicating that more labor can diversify agricultural outputs or increase income, thus enabling a more varied diet (53).

**Table 4:** Heterogeneity analysis based on labor number.

**Variable**	**E**		**CFPS**		**CHEI**	
	**Labor number**		**Labor number**		**Labor number**	
	≤**2**	>**2**	≤**2**	>**2**	≤**2**	>**2**
Age < 18	0.001	0.002	−0.001	0.002	−0.021	−0.019
	(0.001)	(0.002)	(0.004)	(0.006)	(0.025)	(0.031)
Ratio ≥ 65	−0.001^***^	0.000	−0.001	−0.000	−0.018^***^	−0.021
	(0.000)	(0.002)	(0.001)	(0.005)	(0.006)	(0.032)
Income	0.015^**^	0.019^**^	0.019	0.033	0.155	0.132
	(0.006)	(0.008)	(0.017)	(0.026)	(0.113)	(0.161)
Controlled	Yes	Yes	Yes	Yes	Yes	Yes
County FE	Yes	Yes	Yes	Yes	Yes	Yes
Constant	0.765^***^	0.247	1.585^***^	0.257	21.518^***^	3.473
	(0.160)	(0.304)	(0.541)	(0.961)	(3.772)	(7.041)
N	668	411	668	411	668	411

Robust Standard errors in parentheses. ^*^ *p* < 0.10, ^**^ *p* < 0.05, ^***^ *p* < 0.01.

In contrast, the proportion of elderly members is significantly negatively associated with E and adherence to the CHEI, regardless of labor size. This could suggest that the presence of older members might entail a dietary pattern that is less diverse and less aligned with dietary guidelines due to age-related needs and preferences, as seen in studies such as Castro et al. (54). Interestingly, in households with more labor, the negative impact on the CHEI is diminished, suggesting that a larger working-age population may alleviate some of the dietary restrictions commonly faced by the elderly through better household resource allocation. The variable of household income exhibits a positive relationship with all three dietary health measures, particularly in households with more labor, reinforcing the idea that greater economic resources lead to better diet quality, a finding that resonates with the conclusions of Strauss and Thomas (55).

#### 4.2.3 Pension

Table 5 is divided into results based on low and high levels of pension income and examines their association with healthy diet. For households with a lower pension income, the impact on the E is positive for younger household members (Age < 18) but negative for older household members (Age ≥ 65). This may suggest that in lower-income settings, households prioritize dietary variety for younger members, which could be due to the heightened nutritional requirements for growth and development. However, the negative impact on older members may reflect fixed incomes limiting diverse food purchases, a trend identified in prior studies examining economic constraints on dietary choices among the elderly (56). In contrast, for households with a higher pension income, we see a stark difference. The Age < 18 has an even stronger positive effect on the Entropy Index of Dietary Diversity, indicating that additional financial security may further enable the provision of varied diets for younger members. The Age ≥ 65, however, is associated with a slight increase in the Chinese Healthy Eating Index, suggesting that greater pension income might allow older members to adhere more closely to dietary guidelines, a finding that echoes the work by Grewal et al. (57) on the positive correlation between pension wealth and health outcomes. The effect of income is consistent across different levels of pension income and positive for all three health diet indicators, reinforcing the notion that higher income contributes to better dietary health, as established by Case (58). Overall, the heterogeneity revealed by pension income levels offers important insights into the interplay between economic security and dietary habits in rural China. It underscores the necessity for nuanced policy frameworks that address not only general income levels but also the distribution and adequacy of pension wealth to improve dietary outcomes across different age groups within rural households.

**Table 5:** Heterogeneity analysis based on pension.

**Variable**	**E**		**CFPS**		**CHEI**	
	**Ln(pension)**		**Ln(pension)**		**Ln(pension)**	
	**Low**	**High**	**Low**	**High**	**Low**	**High**
Age < 18	0.002^**^	0.003	−0.001	0.001	0.001	0.122^*^
	(0.001)	(0.003)	(0.003)	(0.015)	(0.016)	(0.064)
Age ≥ 65	−0.001^*^	−0.001	−0.001	−0.001	−0.015^**^	0.007
	(0.000)	(0.001)	(0.001)	(0.003)	(0.007)	(0.012)
Income	0.017^***^	0.031	0.023	0.091	0.138^*^	−0.001
	(0.004)	(0.026)	(0.014)	(0.089)	(0.078)	(0.476)
Controlled	Yes	Yes	Yes	Yes	Yes	Yes
County FE	Yes	Yes	Yes	Yes	Yes	Yes
Constant	0.736^***^	0.405	1.154^**^	0.789	18.613^***^	14.236^*^
	(0.148)	(0.420)	(0.526)	(1.666)	(3.679)	(8.139)
N	963	116	963	116	963	116

Robust Standard errors in parentheses. ^*^ *p* < 0.10, ^**^ *p* < 0.05, ^***^ *p* < 0.01.

#### 4.2.4 Food market density

Table 6 provides a nuanced view of how food market density interacts with family composition and income to influence dietary health indicators in rural China. The analysis is divided by low and high food market density areas, comparing their effects on healthy diet. For households in areas with low food market density, we observe that the proportion of members under 18 (Age < 18) is positively associated with the Entropy Index of Dietary Diversity. This may suggest that even in areas with fewer food purchasing options, households prioritize a varied diet for their younger members. However, the proportion of elderly members (Age ≥ 65) is significant negative on E and CHEI in low food market density group. The potential reason for this is that elderly individuals in regions with limited food market availability have reduced food accessibility, which hinders their ability to obtain a diverse range of foods (59).

**Table 6:** Heterogeneity analysis based on food market density.

**Variable**	**E**		**CFPS**		**CHEI**	
	**Food market density**		**Food market density**		**Food market density**	
	**Low**	**High**	**Low**	**High**	**Low**	**High**
Age < 18	0.003^***^	0.001	0.000	0.000	0.011	0.002
	(0.001)	(0.002)	(0.003)	(0.004)	(0.022)	(0.025)
Age ≥ 65	−0.001^**^	−0.000	−0.002	−0.001	−0.020^**^	−0.007
	(0.000)	(0.000)	(0.001)	(0.002)	(0.007)	(0.007)
Income	0.014^***^	0.021^**^	0.020	0.024	0.068	0.227
	(0.005)	(0.009)	(0.017)	(0.025)	(0.090)	(0.159)
Controlled	Yes	Yes	Yes	Yes	Yes	Yes
County FE	Yes	Yes	Yes	Yes	Yes	Yes
Constant	1.029^***^	0.708^***^	2.388^***^	0.345	23.309^***^	13.005^**^
	(0.206)	(0.170)	(0.519)	(0.886)	(4.881)	(5.191)
N	689	390	689	390	689	390

Robust Standard errors in parentheses. ^*^ *p* < 0.10, ^**^ *p* < 0.05, ^***^ *p* < 0.01.

Income exhibits a positive relationship with all dietary health indicators in both low and high food market density areas, with the impact being more pronounced in high-density areas. This indicates that income increases can lead to more significant improvements in dietary health where food options are more abundant, which is consistent with economic theories linking income growth with improved diet quality (9).

#### 4.2.5 Land area

The results of Table 7 indicate that for households with smaller land sizes [Low Ln(Land)], the proportion of household members under 18 (Age < 18) is positively associated with E. This positive association may reflect a greater need for nutritional variety among younger household members, a finding consistent with research stressing the importance of adequate nutrition for growth and development (60). For larger landholdings, the relationship between Age < 18 and dietary measures is less clear, potentially due to different agricultural practices or resource allocations that come with larger landholdings. For older household members (Age ≥ 65), there is a consistent negative association with the CHEI across land size categories, emphasizing the challenges faced by the elderly in achieving a diet that meets health guidelines. The negative coefficients are larger for households with more land, which could suggest that despite the potential for greater agricultural production, dietary quality does not necessarily improve for the elderly. This might be attributed to the focus on agricultural output over nutritional diversity or possible health limitations in older age, which may limit consumption variety despite availability (61). Income levels show a positive and significant impact on the E across both land size categories, reinforcing the understanding that higher income enables access to a more qualified diet.

**Table 7:** Heterogeneity analysis based on food land area.

**Variable**	**E**		**CFPS**		**CHEI**	
	**Ln(Land)**		**Ln(Land)**		**Ln(Land)**	
	**Low**	**High**	**Low**	**High**	**Low**	**High**
Age < 18	0.002^*^	0.001	0.001	−0.005	0.010	−0.016
	(0.001)	(0.001)	(0.003)	(0.004)	(0.020)	(0.034)
Age ≥ 65	−0.001^**^	−0.001	−0.002	−0.002	−0.018^**^	−0.015
	(0.000)	(0.001)	(0.001)	(0.002)	(0.007)	(0.013)
Income	0.018^***^	0.015^***^	0.032	0.010	0.169	0.042
	(0.006)	(0.005)	(0.019)	(0.020)	(0.125)	(0.107)
Controlled	Yes	Yes	Yes	Yes	Yes	Yes
County FE	Yes	Yes	Yes	Yes	Yes	Yes
Constant	0.616^***^	1.016^***^	0.938	0.984	15.802^***^	24.603^***^
	(0.135)	(0.353)	(0.595)	(1.046)	(3.605)	(8.255)
N	719	360	719	360	719	360

Robust Standard errors in parentheses. ^*^ *p* < 0.10, ^**^ *p* < 0.05, ^***^ *p* < 0.01.

### 4.3 Impact of family composition and income on the consumption of each food group

Table 8 presents the impact of family composition and income on different types of food consumption. Starting with family composition, the proportion of household members under 18 (Age < 18) has a significantly negative association with the consumption of total grains and vegetables, yet it positively influences the consumption of fruits and meat & poultry. This could be indicative of younger households prioritizing more diverse and possibly nutrient-dense foods over traditional staples like grains. The positive effect on meat & poultry consumption aligns with literature that recognizes increased dietary diversity with higher nutrient requirements during growth periods (62).

**Table 8:** Impact of family composition, income on consumption of different food groups.

**Variable**	**Total Grains**	**Vegetables**	**Fruits**	**Meat and Poultry**	**Eggs**	**Aquatic products**	**Dairy**	**Nuts**
Age < 18	−1.195^***^	−1.217^**^	0.060	0.677^***^	−0.046	−0.145	−0.024	−0.012
	(0.435)	(0.519)	(0.556)	(0.228)	(0.124)	(0.161)	(0.117)	(0.027)
Age ≥ 65	−0.256	−0.150	−0.796^***^	−0.078	−0.040	−0.101^*^	−0.030	0.007
	(0.165)	(0.197)	(0.211)	(0.086)	(0.047)	(0.061)	(0.045)	(0.010)
Income	0.526	−1.520	8.003^***^	7.268^***^	1.694^***^	2.016^**^	1.557^**^	−0.081
	(2.274)	(2.718)	(2.910)	(1.191)	(0.650)	(0.843)	(0.615)	(0.141)
Controlled	Yes	Yes	Yes	Yes	Yes	Yes	Yes	Yes
County FE	Yes	Yes	Yes	Yes	Yes	Yes	Yes	Yes
Joint significant test of Age ≥65				19.84^***^				
Joint significant test of Income				57.56^***^				
N	1,079	1,079	1,079	1,079	1,079	1,079	1,079	1,079

Robust Standard errors in parentheses. ^*^ *p* < 0.10, ^**^ *p* < 0.05, ^***^ *p* < 0.01.

For households with a larger proportion of elderly members Age ≥ 65), there's a substantial positive association with fruit consumption, which may suggest a dietary preference or need for foods that are easier to consume and digest for older adults, as noted by Gallego et al. (63) in their study on aging and diet diversification. Conversely, this demographic has a negative effect on aquatic product consumption, potentially due to the higher cost or preparation requirements of these foods, as the elderly may face financial or functional limitations (64).

Income has a mixed impact across food types. Notably, higher income significantly increases the consumption of meat & poultry, dairy, and fruits, which are often more expensive food items, implying that financial capacity enables the purchase of a more diverse range of foods, including protein-rich items. This is supported by the income elasticity of food consumption found in Brewis et al. (65), which suggests that as incomes rise, households tend to spend a greater proportion on non-staple food items.

The joint significance tests for the ratios and income indicate strong combined effects of these variables on food consumption patterns, highlighting the interconnectedness of age demographics and economic capacity in dietary choices. Overall, these findings point to the critical role that family structure and economic status play in shaping dietary behavior, with implications for nutritional interventions and policies aiming to improve diet quality among different demographic groups within rural households.

### 4.4 Predicting the changes of healthy diet in China

**Figure 3:**
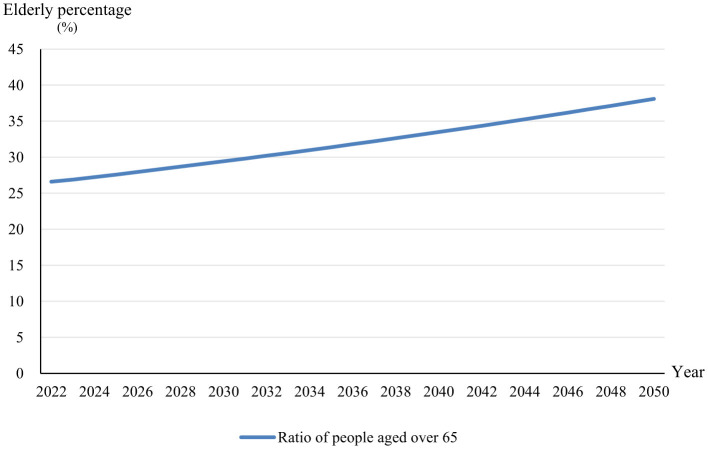
Prediction of changes in proportion of elderly people in China.

Figure 3 depicts a forecasted increase in the proportion of the elderly population, specifically those aged 65 and above, in China over the period from 2023 to 2050. It presents a linear upward trajectory, reflecting a steady growth rate. The slope of the line indicates a moderate, yet consistent, year-over-year increase at an average annual rate of 1.03%, as reported by the United Nations (66). This gradual incline suggests a notable shift toward an older population, with the percentage of elderly individuals rising from just over 20% to approaching 40% over the 27-year span. The implications of this demographic change are significant, potentially impacting various sectors including healthcare, pension systems, labor markets, and economic growth. The United Nations' data thus offers a crucial insight into the long-term population trends of China, highlighting the need for strategic planning in response to an aging society.

**Figure 4:**
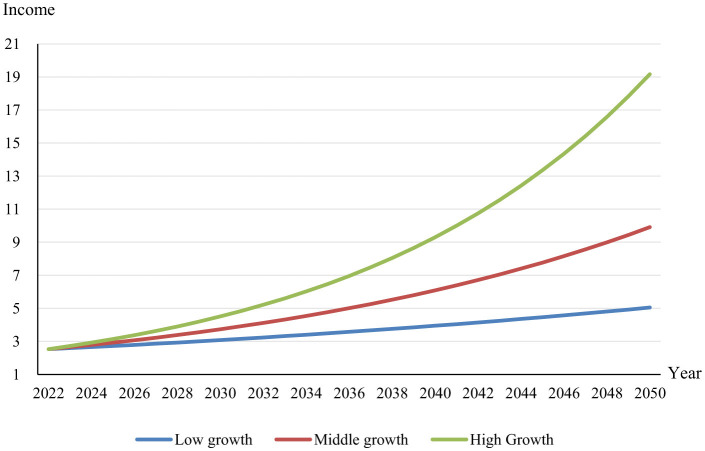
Prediction of changes in per capita income in China. The unit of income is the thousand Yuan.

In Figure 4, we observe three projected income growth trajectories from 2023 to 2050. These trajectories differ by their respective annual growth rates: a low growth scenario at 2.5%, a medium growth scenario at 5%, and a high growth scenario at 7.5%. The low growth path, represented by the blue line, exhibits a moderate upward slope, indicating gradual increases in income levels over time. The orange line, corresponding to the medium growth scenario, depicts a steeper ascent, suggesting more robust income improvements. The most pronounced increase is shown by the yellow line, indicative of the high growth scenario, demonstrating significant elevation over the period and substantial income augmentation. The graph highlights the compound effect of varying growth rates on economic outcomes, illustrating how even modest differences can cause significant divergence in long-term income levels. Notably, the high growth scenario underscores the potential for rapid income escalation, influencing consumption patterns significantly, possibly enhancing or hindering sustainable food objectives depending on the types of food consumed. Additionally, the shifting demographic structure toward an aging population may alter food demand, reducing meat consumption and increasing intake of fruits and vegetables, further impacting sustainability. Conversely, the low growth scenario might indicate more conservative economic progress, necessitating targeted policy interventions to encourage sustainable dietary practices and accommodate the changing food preferences resulting from demographic shifts.

**Figure 5:**
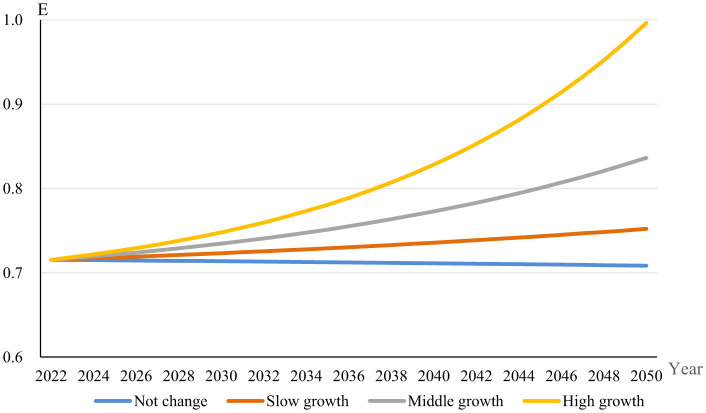
Entropy index trends in China.

Figure 5 presents projected changes in the Entropy Index of dietary diversity based on different income growth levels extending to the year 2050. The Entropy Index, as an indicator of dietary diversity, measures nutritional adequacy and food consumption patterns. The blue line represents a scenario with no income growth, suggesting a stable Entropy Index over time and implying limited scope for dietary improvements without income increases. The orange line, illustrating a low-income growth scenario of 2.5%, indicates a modest upward trajectory in dietary diversity, demonstrating that even slight income growth can facilitate greater dietary variety, potentially aligning with sustainable food goals through improved nutritional outcomes. The gray line, representing a middle-income growth scenario at 5%, shows a more pronounced rise in the Entropy Index, emphasizing a stronger linkage between increased economic resources and diversified diets, thus offering potential support for sustainability objectives if dietary shifts favor environmentally sustainable food choices. Finally, the green line, corresponding to high-income growth at 7.5%, exhibits the most substantial increase, underlining the significant potential for robust income growth to enhance dietary diversity and, consequently, influence sustainable food consumption positively or negatively based on specific food preferences and consumption behaviors. These patterns are aligned with economic theories that posit increased economic capacity allows households to diversify their diets, thus potentially enhancing diet quality and sustainability (25).

**Figure 6:**
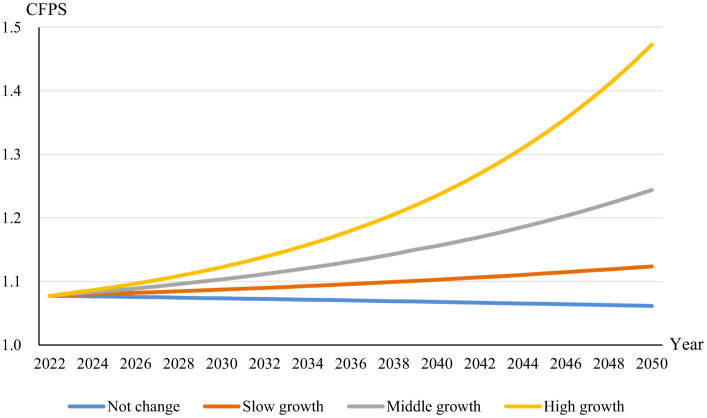
CFPS trends in China.

Figure 6 illustrates the expected trajectory of the CFPS, an index measuring adherence to recommended dietary patterns, over the period from 2023 to 2050 under varying income growth conditions. The blue line represents the scenario where income does not change, remaining flat over the years. Under this condition, the CFPS is predicted to remain essentially static, suggesting that without economic growth, there is no improvement in the adherence to dietary guidelines. In the case of slow income growth, at a rate of 2.5%, shown by the orange line, there is a gradual upward trend in the CFPS. This indicates some improvement in dietary quality, albeit at a conservative pace, implying that even slight economic growth can positively affect diet quality. The gray line reflects a middle-income growth scenario at 5%. Here, we see a more pronounced increase in the CFPS, suggesting that moderate economic growth could substantially influence the ability of individuals or households to comply with dietary recommendations. Finally, the green line depicts the CFPS under a scenario of high-income growth at 7.5%. The steep ascent of this line suggests a strong positive relationship between significant income growth and dietary quality, implying that higher economic growth rates could lead to considerable improvements in dietary adherence.

**Figure 7:**
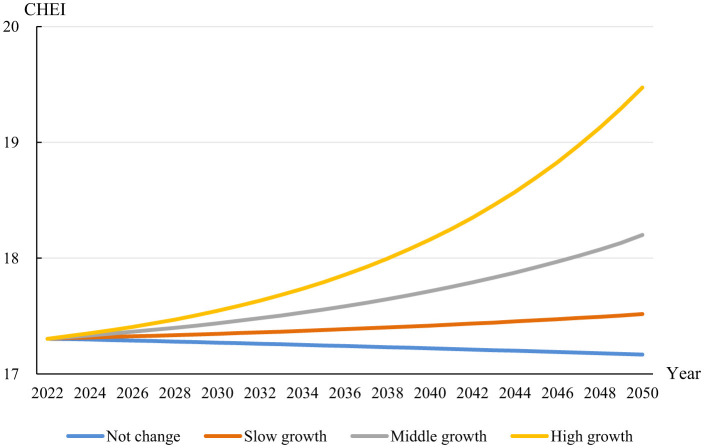
CHEI index trends in China.

Figure 7 delineates the forecasted progression of the CHEI from 2023 to 2050 across different income growth scenarios. The CHEI serves as a gauge for assessing the conformity of dietary patterns to established nutritional standards in China. In the static income scenario, represented by the blue line, the CHEI demonstrates negligible variation over the projection period, implying a direct correlation between stagnant income levels and stable dietary habits, potentially posing challenges to achieving sustainable food objectives. Under the slow income growth scenario, characterized by an annual increase of 2.5% and represented by the orange line, the CHEI shows a modest ascent. This indicates that incremental economic improvements can yield gradual enhancements in dietary adherence, potentially aiding progress toward sustainable dietary goals. A more noticeable upward trajectory is evident in the gray line, representing a moderate-income growth rate of 5%, highlighting a stronger response of dietary quality to economic advances. Such improvements can increase the affordability and consumption of nutritious and environmentally sustainable food choices. The green line illustrates the most optimistic outcome, corresponding to a high-income growth rate of 7.5%. The steep gradient of this line underscores a strong link between significant economic growth and substantial dietary advancements, reinforcing that robust income growth could drive major improvements in adherence to healthy and sustainable eating practices (67).

### 4.5 Robustness check

To further validate the robustness of our baseline forecasting models, we applied the Least Absolute Shrinkage and Selection Operator (LASSO) regression (68). Specifically, LASSO estimates the coefficient vector β by minimizing the following objective function:


(3) $$\begin{array}{c} \underset{\beta }{\min}\{\sum_{i=1}^{N}{\left(y_{i}-X_{i}\beta \right)}^{2}+\lambda \sum_{j=1}^{p}\left|\beta_{j}\right|\} \end{array}$$

where *y*_*i*_ is the dietary health outcome for household *i*, *X*_*i*_ is the *p*-dimensional vector of candidate predictors, β is the vector of coefficients, *N* is the sample size, *p* is the number of predictors, and λ is the non-negative penalty parameter that controls the degree of shrinkage. By combining variable selection and coefficient regularization in a single step, LASSO reduces multicollinearity and overfitting, often achieving better predictive accuracy on data (69, 70). Recent studies have highlighted its effectiveness in high-dimensional settings (71, 72). We used the glmnet package with 10-fold cross-validation (73, 74) to choose the optimal λ, and Appendix Figures A2–A4 show that our main findings remain robust when only the most influential predictors are retained.

The results from the LASSO regression largely corroborate our initial findings, demonstrating consistency in the predicted impacts of family composition and income on dietary health metrics. The robustness check confirms that increases in household income continue to exhibit a significant positive influence on dietary diversity and quality, as measured by E, CFPS, and CHEI. Similarly, the adverse effects associated with a higher proportion of elderly household members were reaffirmed, underlining the challenges posed by aging demographics on achieving a healthy diet.

These consistent findings across different analytical methodologies enhance the credibility of our conclusions and support the robustness of our original econometric models. Consequently, the policy recommendations advocating targeted economic and nutritional interventions in rural Chinese populations are further validated. This robust evidence underscores the importance of developing policy measures that simultaneously address economic constraints and demographic changes, thereby effectively improving dietary patterns and supporting broader public health and sustainability objectives (75).

## 5 Conclusions

A healthy diet is essential for balanced nutrition (76) and dietary diversity is closely linked to sustainable development (77). This paper investigates how family composition and income influence healthy dietary patterns among rural households. To enhance the robustness of our findings, we utilize three distinct indicators of dietary health. Our results reveal that a higher proportion of elderly household members is consistently associated with poorer performance on healthy diet measures, whereas greater per-capita income reliably improves dietary diversity, guideline adherence, and overall diet quality. Specifically, we observe that aging populations tend to decrease household consumption of fruits and aquatic products, whereas higher income levels encourage increased intake of protein-rich foods, including meat, poultry, eggs, dairy products, and aquatic products. Heterogeneity analyses reveal that the adverse impact of a high elderly share is most pronounced in low-income households and in areas with sparse food market density, whereas more labor numbers, larger land area and higher pension incomes attenuate this negative effect. Predictive analyses indicate that a continued rise in the share of elderly household members will further erode dietary quality, although higher household income may partially mitigate these adverse effects.

This study contributes to the understanding of sustainable food consumption by identifying key demographic and economic factors that shape rural dietary patterns. The findings underscore the importance of integrated approaches that consider income dynamics, demographic trends such as population aging in promoting healthy sustainable diets. Elucidating these determinants provides a foundation for future research into rural dietary behaviors and their broader sustainability implications and aligns directly with the United Nations SDGs, particularly SDG 2 on achieving zero hunger through improved food security and dietary diversity, SDG 3 on ensuring healthy lives and wellbeing by guiding nutrition-sensitive interventions for aging populations.

The findings of this study offer actionable guidance for rural nutrition policy not only in China but also in other developing countries facing similar demographic and economic challenges. First, the robust positive link between household income and dietary health implies that interventions such as microfinance programs, support for smallholder farmers and market integration initiatives can be effective levers for improving diet quality (78). Second, with rising shares of elderly in the rural population, investments in age friendly infrastructure, including community dining facilities, accessible transport to markets and clinics and elder support services, can help mitigate the nutritional risks associated with an aging demographic (79). Third, for economically disadvantaged households, direct nutrition assistance through targeted subsidies or vouchers for fruits, vegetables, protein-rich food and other nutrient dense foods can bridge affordability gaps and promote healthier consumption patterns. By adapting these strategies to local institutional contexts, cultural preferences and market structures, policymakers in other low and middle income countries can design more effective interventions, thereby advancing global commitments under SDG 2 (Zero Hunger) and SDG 3 (Good Health and Well Being).

This paper also has limitations. Firstly, the analysis relies on cross-sectional data, which cannot eliminate the impact of inherent factors at the family or individual level. Secondly, the sample selection in this study covers three provinces in China, and conclusions drawn from using a more nationally distributed sample will be more representative. Therefore, it is recommended that future studies collect a more comprehensive panel of data to gain a better understanding of the healthy diet status among rural residents in China.

## Data Availability

The raw data supporting the conclusions of this article will be made available by the authors, without undue reservation.
