# Supplementary material for: Family composition, income, and healthy diet in rural China: evidence from three provinces
Source: Front Nutr. 2025 Jun 4;12:1608024. doi: 10.3389/fnut.2025.1608024 (PMC12173862; doi:10.3389/fnut.2025.1608024)
Supplement: Supplementary file 1 [file Image_1.pdf]

Appendix

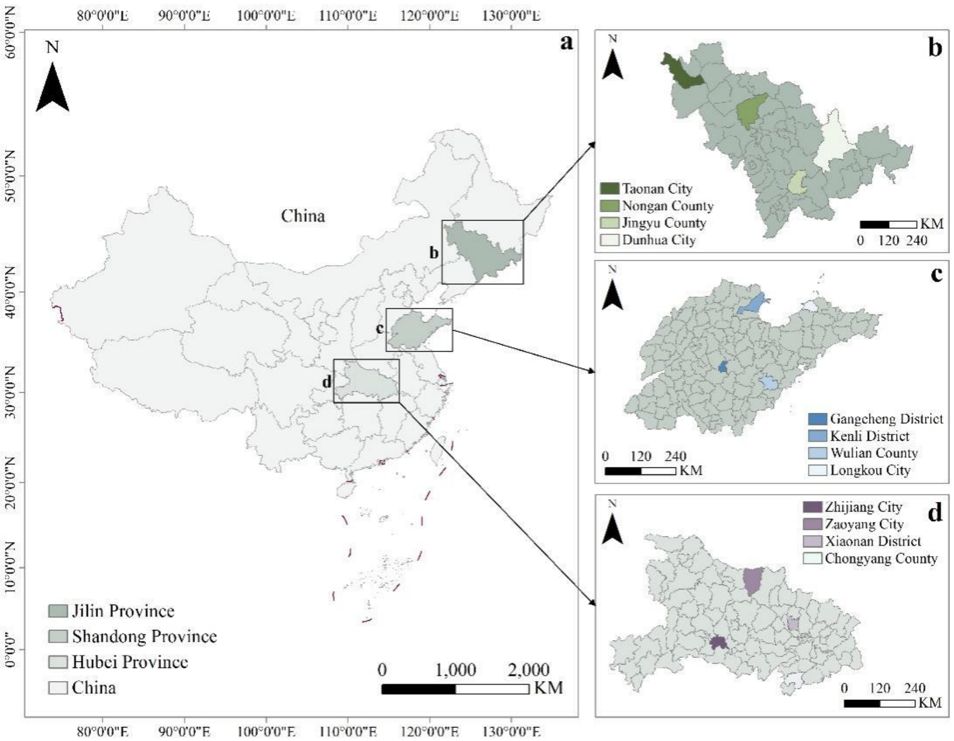


Figure A1 Geographic distribution of sampled provinces and counties

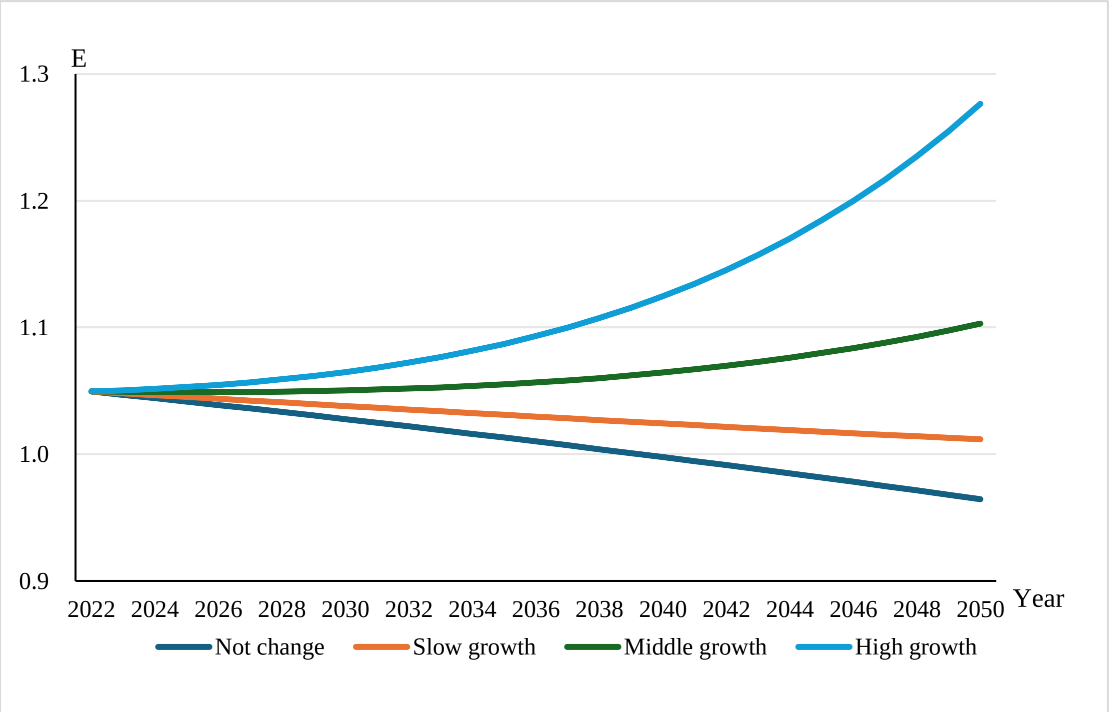


Figure A2 Entropy index trends in China by LASSO

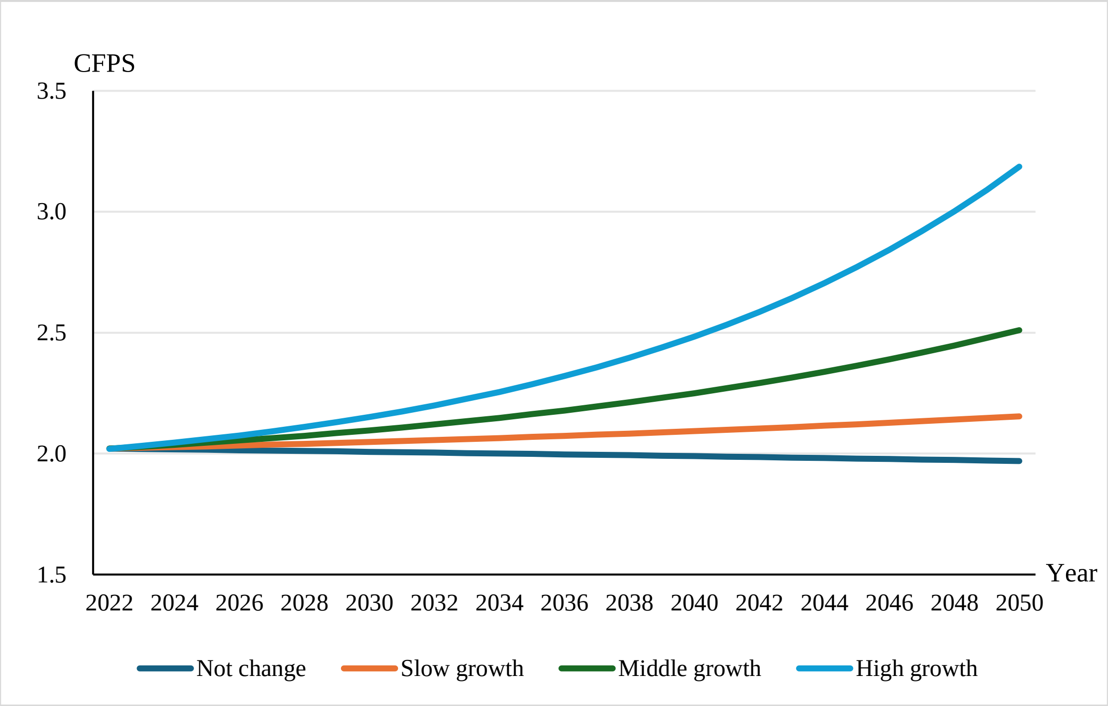


Figure A3 CFPS trends in China by LASSO

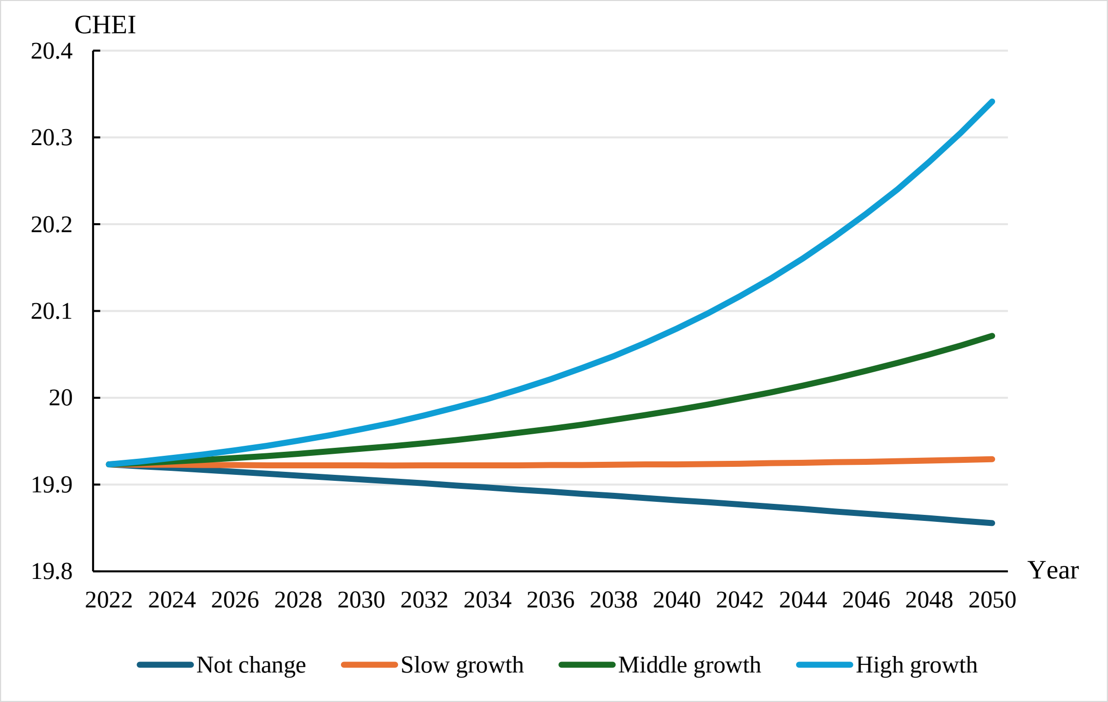


Figure A4 CHEI trends in China by LASSO
